# CHROMIXS: automatic and interactive analysis of chromatography-coupled small-angle X-ray scattering data

**DOI:** 10.1093/bioinformatics/btx846

**Published:** 2017-12-28

**Authors:** Alejandro Panjkovich, Dmitri I Svergun

**Affiliations:** European Molecular Biology Laboratory, Hamburg Outstation, EMBL c/o DESY, Hamburg, Germany

## Abstract

**Summary:**

Size-exclusion chromatography (SEC) coupled to small-angle X-ray scattering (SAXS), also known as inline SEC-SAXS, is being increasingly used for the structural analysis of biological macromolecules, complexes and mixtures in solution. A single SEC-SAXS run generates thousands of individual SAXS profiles from the eluting solute and their analysis requires a correct identification of buffer and sample regions, a rather laborous task. We present CHROMIXS (as in CHROMatography Inline X-ray Scattering), a program for rapid reduction and analysis, both automatically and interactively, of SEC-SAXS data.

**Availability and implementation:**

CHROMIXS is freely available to academic users as part of the ATSAS software suite (www.embl-hamburg.de/biosaxs/download.html).

## 1 Introduction

Small-angle X-ray scattering (SAXS) is becoming a standard tool for the structural characterization of biological macromolecules in solution (Vestergaard, 2016). Using a small (below 1 mg) quantity of purified material, SAXS provides the overall size and overall structure of macromolecules at a resolution of about ∼ 10–20 Å. In a typical batch SAXS experiment, scattering from the sample and buffer are measured separately for subsequent subtraction. Cases that are particularly difficult to assess and interpret, such as transient complexes or mixtures, highly benefit from directly coupling the elution on a size-exclusion chromatography column (SEC) to the SAXS sample exposure unit (SEC-SAXS). These experiments can separate the individual components and provide their individual SAXS patterns. During a SEC-SAXS experiment, scattering curves (frames) are recorded continuously every (few) second(s) during the elution of the sample (usually ∼ 30–60 min). The experiment can be coupled to ultra-violet and additional detectors to further characterize the sample biophysically (Jeffries *et al.*, 2016). Instead of a handful of recorded scattering curves as in batch SAXS, a SEC-SAXS experiment produces thousands of unsubtracted curves among which optimal buffer and sample regions need to be identified. Until recently, the reduction and analysis of SEC-SAXS datasets was cumbersome and lengthy, even for experts using a combination of manual processing and *ad-hoc* scripts.

**Fig. 1.**
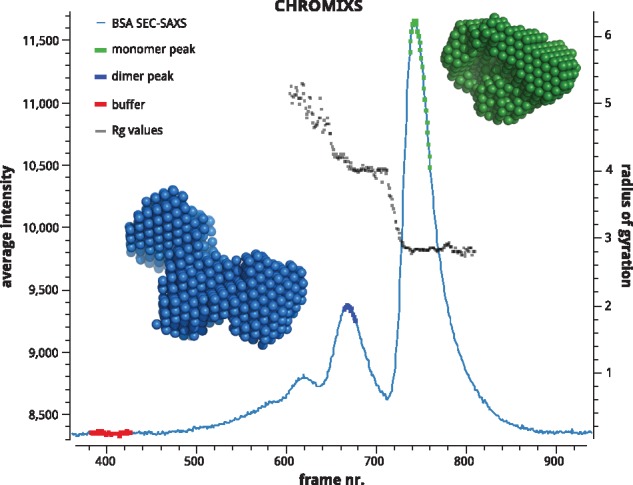
CHROMIXS plot showing integrated intensities (light blue) versus time (frame number) for a SEC-SAXS run on bovine serum albumin (BSA) at EMBL P12 BioSAXS beamline. Two sample peaks (green and blue) and buffer region (red) have been predicted automatically by CHROMIXS, with the R_*g*_ values (black) estimated for the relevant regions. Bead models corresponding to *ab-initio* shape reconstructions based on the subtracted data are shown in green for the monomer peak and in blue for the dimer peak (Color version of this figure is available at *Bioinformatics* online.)

Here, we present CHROMIXS (as in CHROMatography Inline X-ray Scattering), a user-friendly tool that allows rapid visualization and reduction, both automatically and interactively, of SEC-SAXS datasets. CHROMIXS streamlines the work of expert users and it also allows non-experts to quickly evaluate the results of a SEC-SAXS run. Importantly, CHROMIXS can work fully unsupervised in automatic mode, identifying sample and buffer regions from the elution profile to produce a final subtracted SAXS curve. The graphical interface provides additional functionality, such as automatic estimation of the radius of gyration (R_*g*_) and molecular weight values, with options to export both raw data and scalable vector graphics (SVG) for the preparation of publication quality figures (Fig. 1).

## 2 Description

CHROMIXS is written in C++ and relies on *Qt* and *Qwt* for the cross-platform graphical interface, on *libsaxsdocument* for input/output formats and partially on ATSAS specific library functions for data processing (Franke *et al.*, 2017) (some functions were re-written to optimize SEC-SAXS data processing). The program is cross-platform, running in Linux, Mac OS X and Windows operating systems. Although multiple tools exist for the analysis of SAXS profiles, CHROMIXS is implemented specifically for the reduction and analysis of SEC-SAXS data, allowing a series of optimizations related to data loading, visualization and automatic processing.

Upon startup, CHROMIXS rapidly parses all frame files successively recorded during SEC-SAXS experiment and presents the user with a graphical preview of the elution curve as averaged scattering intensity versus time (frame number) as shown in (Fig. 1). By default, intensities are averaged for each frame within the range 0.1 to 0.8 nm^–^^1^ of momentum transfer s=4π sin ⁡(θ)/λ, where 2θ is the scattering angle and *λ* corresponds to the X-ray wavelength. The overall noise level is estimated according to the variation in average intensity between frames.

The user can define sample and buffer regions using the mouse through CHROMIXS’ graphical interface. Commonly, the sample region corresponds to the tip of the elution peak of interest, while matching buffer is expected to elute in the flat region just before the sample begins to elute. Alternatively, CHROMIXS’ automatic sample prediction function can be called, which is a peak-finder implementation based on the derivatives of the elution profile curve adjusted by the initial noise estimation.

Once a sample region has been defined, a buffer region may be selected manually or automatically. The automatic buffer prediction procedure uses an average over a sliding window of *N* consecutive frames (the same number as in the selected sample) to find low intensity and continuous regions in the elution profile. Each putative buffer region found is subtracted from the sample signal and the subtracted curves are scored using the ‘quality’ measure of the *autorg* ATSAS routine (Franke *et al.*, 2017), which accounts for the amount of negative intensities, length and residuals obtained upon the R_*g*_ determination (so-called Guinier fit). The region with the best quality score after subtraction is selected as the optimal buffer region.

Once the buffer and sample regions have been defined, single buffer frames are subtracted from sample frames one by one, scaled and averaged to produce the final subtracted curve. This approach allows for statistically rigorous estimation and propagation of the associated errors (manuscript in preparation).

For publication-quality figures, CHROMIXS can export plots in SVG open standard format. The intensity versus frame number curve and R_*g*_ values estimated by CHROMIXS can also be exported as raw data in CSV format, for customized plotting or treatment. Alternatively, data can be saved in CHROMIXS’ native format, to continue the analysis later. The users can also utilize the ‘open selected frames in Primus’ menu to redirect selected buffer or sample frames to *PRIMUS/qt* program for the detailed analysis of individual frames, including statistical comparisons (Franke *et al.*, 2017).

CHROMIXS can run completely unsupervised for high-throughput processing without activating the graphical interface. In this fully automatic mode, CHROMIXS loads all frames in the data directory, predicts sample(s) and buffer regions and produces subtracted curve(s). Details of the selection and results are written as metadata in the output file for future reference. An automatic reduction of a bovine serum albumin (BSA) SEC-SAXS dataset consisting of 1800 SAXS one-dimensional frames (Fig. 1) takes a few seconds on a standard desktop computer.

## 3 Concluding remarks

CHROMIXS allows for a convenient manual or automated reduction of SEC-SAXS runs with well resolved fractions (i.e. baseline separated sample elution peaks). In more intricate cases, CHROMIXS may help in diagnosing and preparing the data for further processing by other programs (as these require buffer frames to be manually defined or subtracted beforehand). For example, overlapping peaks may be decomposed by *BioXTAS RAW* (Hopkins *et al.*, 2017) or *UltraScan-SOMO* (Brookes *et al.*, 2016), the latter also providing a function to ease capillary fouling issues.

Currently, CHROMIXS is being routinely used at the EMBL Hamburg P12 BioSAXS beamline, where an increasing percentage of measurements are performed in SEC-SAXS mode. CHROMIXS reads plain ASCII files in and it was tested on the experimental data generated by other SAXS beamlines (e.g. from synchrotrons ESRF, Diamond and Soleil). In addition to a fully automatic SEC-SAXS processing function, CHROMIXS provides a responsive and easy-to-use interface, optimal errors propagation and experiment quality assessment together with thorough data and figure export features.
